# Early motion and directed exercise (EMADE) versus usual care post ankle fracture fixation: study protocol for a pragmatic randomised controlled trial

**DOI:** 10.1186/s13063-018-2691-7

**Published:** 2018-05-31

**Authors:** Paul A. Matthews, Brigitte E. Scammell, Arfan Ali, Timothy Coughlin, Jessica Nightingale, Tanvir Khan, Ben J. Ollivere

**Affiliations:** 1 Academic Orthopaedics, Trauma and Sports Medicine, Division of Rheumatology, Orthopaedics and Sports Medicine, University of Nottingham, Nottingham, NG7 2UH UK; 2 Nottingham University Hospitals NHS Trust, Arthritis Research UK Centre for Sport, Exercise and Osteoarthritis, Nottingham, NG7 2UH UK; 3 Nottingham University Hospitals NHS Trust, Queen’s Medical Centre, Nottingham, NG7 2UH UK; 4 Division of Physiology, Pharmacology and Neuroscience, School of Life Sciences, University of Nottingham, Nottingham, NG7 2UH UK

**Keywords:** Ankle, Fracture, Early rehabilitation, Exercise, Physiotherapy, Health economics

## Abstract

**Background:**

Following surgical fixation of ankle fractures, the traditional management has included immobilisation for 6 weeks in a below-knee cast. However, this can lead to disuse atrophy of the affected leg and joint stiffness. While early rehabilitation from 2 weeks post surgery is viewed as safe, controversy remains regarding its benefits. We will compare the effectiveness of early motion and directed exercise (EMADE) ankle rehabilitation, against usual care, i.e. 6 weeks’ immobilisation in a below-knee cast.

**Method/design:**

We have designed a pragmatic randomised controlled trial (p-RCT) to compare the EMADE intervention against usual care. We will recruit 144 independently living adult participants, absent of tissue-healing comorbidities, who have undergone surgical stabilisation of isolated Weber B ankle fractures. The EMADE intervention consists of a non-weight-bearing progressive home exercise programme, complemented with manual therapy and education. Usual care consists of immobilisation in a non-weight-bearing below-knee cast. The intervention period is between week 2 and week 6 post surgery. The primary outcome is the Olerud and Molander Ankle Score (OMAS) patient-reported outcome measure (PROM) at 12 weeks post surgery. Secondary PROMs include the EQ-5D-5 L questionnaire, return to work and return to driving, with objective outcomes including ankle range of motion. Analysis will be on an intention-to-treat basis. An economic evaluation will be included.

**Discussion:**

The EMADE intervention is a package of care designed to address the detrimental effects of disuse atrophy and joint stiffness. An advantage of the OMAS is the potential of meta-analysis with other designs. Within the economic evaluation, the cost-utility analysis, may be used by commissioners, while the use of patient-relevant outcomes, such as return to work and driving, will ensure that the study remains pertinent to patients and their families. As it is being conducted in the clinical environment, this p-RCT has high external validity. Accordingly, if significant clinical benefits and cost-effectiveness are demonstrated, EMADE should become a worthwhile treatment option. A larger-scale, multicentre trial may be required to influence national guidelines.

**Trial registration:**

ISRCTN, ID: ISRCTN11212729. Registered retrospectively on 20 March 2017.

**Electronic supplementary material:**

The online version of this article (10.1186/s13063-018-2691-7) contains supplementary material, which is available to authorized users.

## Background

### Background and rationale

Ankle fractures are extremely common, accounting for over 20% of all lower-limb fractures [1] . The AO (Arbeitsgemeinschaft für Osteosynthesefragen) system of open reduction and internal fixation (ORIF) technique, has become the accepted treatment for unstable ankle fractures [2]. The traditional post-surgery management has been based on 6 weeks’ non-weight-bearing, with the ankle immobilised in a below-knee cast.

Detrimental sequelae of this traditional regimen are a combination of joint stiffness, reduced range of motion (ROM), pain, reduced circulation, oedema and muscle atrophy [3–6]. This presentation has been termed ‘fracture disease’ [2, 7, 8] and ‘cast disease’ [7, 9–12]; the term ‘cast disease’ is used through the remainder of this protocol. Attempts to address cast disease can necessitate extended rehabilitation, but even so, not all patients obtain the desired recovery [13]. The necessity of delaying rehabilitation for 6 weeks is being increasingly questioned.

Systematic reviews of previous randomised control trials (RCTs) conclude that early rehabilitation is safe, citing no statistical difference between early rehabilitation and control groups in terms of fixation failure, delayed and non-union and rates of infections. [13–20]. An exception has been where rehabilitation was started immediately after surgery [21], yielding an unacceptable wound infection rate of 66%, in comparison to 16% in the control group. In contrast, delaying rehabilitation until at least 10 days post surgery resulted in a wound infection rate of just 9% [22].

While timing appears to be the single biggest risk factor for iatrogenic wound infections, the reviews identify ambiguity surrounding the evidence supporting effectiveness of early rehabilitation due to risk of methodological bias. For example, underpowered sample sizes and inadequacies in reporting of interventions and results [13–20]. The Cochrane reviewers concluded that while early rehabilitation is mostly safe, there is only ‘limited evidence’ on its effectiveness [13].

Early interventions can be classed broadly as early weight-bearing, early exercises or a combination of both. While NICE [23] recommend further research on early weight-bearing, the impact of early exercise remains an important and under-investigated regimen. Where there has been a focus on exercise as the intervention, it has mostly been limited to range of motion. A comprehensive training programme [24] has shown some benefit over a minimal programme, while in contrast, no supporting evidence was identified by Moseley et al. [4] for the addition of stretches and no supporting evidence for the addition of manual therapy by Lin et al. [25]. However, interventions for these studies commenced after the 6-week period, by which time cast disease would have been established.

We propose that an effective way to address the multifactorial condition of cast disease is the application of a multifactorial physiotherapy intervention. Developed through expert consensus this intervention is based on early motion and directed exercises (EMADE). To determine if the EMADE intervention is effective, it is being assessed against the current usual care for this condition. The EMADE protocol is presented in accordance with Standard Protocol Items: Recommendations for Interventional Trials (SPIRIT) and Template for Intervention Description and Replication (TIDieR) guidelines (see Additional files 1 and 2, respectively).

## Methods/design

### Aim

We aim to establish if, for Weber B ankle fracture patients who have undergone open reduction and internal fixation, whether the EMADE intervention is more effective in reducing symptoms and restoring function than usual care.

#### Primary objective

The primary objective of this study is to test the hypothesis that the early motion and directed exercise (EMADE) physiotherapy intervention, applied in the clinical setting, will perform better than usual care at 12 weeks following operative fixation for Weber B fracture as measured by the Olerud and Molander Score (OMAS) [26].

#### Secondary objectives include

1. A key secondary objective is to determine whether, in this patient group, EMADE will perform better than usual care in the short term (12 weeks post surgery) as measured by the EQ-5D-5 L quality of life measure and the Ankle-Fracture Outcome of Rehabilitation Measure (A-FORM)
2. To determine whether, in this patient group, EMADE will perform better than usual care, in the medium- and long-term (24 and 52 weeks post surgery, respectively), as measured by the OMAS, EQ-5D-5 L and A-FORM
3. To explore the cost-effectiveness of EMADE

### Trial design and study setting

This is a prospective, pragmatic randomised controlled trial (p-RCT) of superiority design, with participants allocated in a 1:1 ratio to either of two parallel groups. The trial is based in the fracture clinic of the Queen’s Medical Centre, Nottingham University Hospitals, Nottingham, UK, with other sites being considered.

## Participants, interventions and outcomes

### Recruitment

The clinical care team will identify potential participants from hospital consultant and theatre lists and notify the researchers of those patients willing to be approached. A researcher will approach the potential participant and inform them of all aspects of the study and provide a written information sheet (available via http://www.isrctn.com/ISRCTN11212729). This states that entry is voluntary and that they are free to withdraw at any time without effect on subsequent care. If appropriate, following screening and following the opportunity to make an informed decision, written consent will be obtained from those willing to be recruited to the study. Consent will be re-confirmed verbally at each stage of the study.

### Eligibility

#### Inclusion criteria

1. Patients with isolated, closed Weber B fractures (AO44-B1, -B2 or -B3) which are stable following open reduction and internal fixation. This includes those requiring syndesmosis stabilisation
2. Patients aged 18 years and over
3. Independently living
4. Capable of independently reading and completing the study paperwork in English

#### Exclusion criteria

1. Inability to provide informed consent, or declining participation
2. Comorbidities: diabetes requiring prescription drugs, non-healing leg/foot ulcers, oral or intravenously administered steroid users, pre-existing ankle arthritis and concurrent or history of significant ipsilateral or contralateral lower limb injury/condition, e.g. prosthesis in lower-limb joints, or neurological disorders
3. At the 2-week clinic visit a patient may be excluded if, based on individual clinical decision, there is notable risk that early wound movement will impede satisfactory healing.
4. Those unable to commit to weekly clinic visits, if assigned to the EMADE intervention group

### Interventions

Pre-operatively and, during the first two post-operative weeks, participants receive identical care consisting of admission to hospital and consultant supervised surgery including management on a standard care pathway. Participants are discharged home in a cast and reviewed 2 weeks following surgery. All participants undergo x-ray post surgery and wound inspection following cast removal during the 2-week review (10 to 19 days post surgery). All participants are non-weight-bearing throughout the study until the 6-week point.

The EMADE intervention is a progressive home exercise programme that includes range-of-motion (ROM) and strengthening exercises, and is conducted by the participant up to six times a day. To be able to conduct the exercises, at the 2-week fracture clinic review, those in the EMADE intervention group are fitted with a removable below-knee cast with Velcro retaining straps.

The EMADE programme starts with light intensity and low daily repetitions, and as the weeks progress, becomes progressively more intensive and repetitive. For example, exercises start resistance-free, and are followed by elastic-exercise-band resistance; while the daily repetitions start twice daily and build up to six times daily. These progressions are taught during weekly face-to-face sessions between the week-2 and week-6 fracture clinic reviews.

During the physiotherapy sessions, participants receive manual therapy consisting of 5 to 10 min of joint and soft-tissue manipulation to the ankle complex. Advice and education is on-going and includes; healing processes, control of pain and swelling, and expectations of fluctuation of pain and swelling. These sessions take part in the recruiting hospital, and are provided by an experienced physiotherapist trained in the application of the EMADE intervention.

To encourage compliance with the EMADE home exercise programme, participants are provided with written and pictorial exercise sheets. These were developed through patient input and include diary sheets, although completion of the diary sheets is neither mandatory nor used in data collection. Participants are not paid to attend, but a basic travel allowance is offered to attend the additional sessions for the study, but not for usual NHS care. Participants in the usual care group are treated in a below-knee cast and remain non-weight-bearing until the 6-week point.

From the 6-week review, all study participants receive the same standard care. This includes removal of cast and, if appropriate following x-ray, weight-bearing as tolerated may commence, along with physiotherapy as required. Protocol deviations will be as per standard care, being based on individual clinical decisions.

### Outcome measures

#### Primary outcome measure

The primary outcome measure is the Olerud and Molander Ankle Score (OMAS) [26] reported at 12 weeks following surgery. The OMAS is a validated ankle-fracture PROM [27, 28] consisting of nine Likert-styled questions; three symptom- and six function-focussed questions. It is scored 0–100; poorest to best, respectively, and has been treated as a continuous scale in the Cochrane ankle fracture review [13] and has been recommended by both Cochrane reviewers [13] and NICE [23].

#### Secondary outcome measures

Secondary outcomes including the OMAS collected at 2 and 6 weeks post surgery, as baseline and end-of-intervention measures, respectively and at 24 and 52 weeks post surgery, as medium- and long-term follow-ups. Other secondary outcomes include: the Ankle-Fracture Outcome of Rehabilitation Measure (A-FORM) [29] and the EQ-5D-5 L [30] PROMs, which are collected at the same time points as the OMAS. Other function-focussed outcomes include the Physical Activity Record Scale (PARS) [31], the Clinical Physical Activity Questionnaire (CPAQ) [32], the use of walking aids, return to work and return to driving. X-ray findings and adverse events are also recorded.

When answering Likert-styled questions within the PROMs, if a participant is unsure which Likert option to select, they are advised to select the poorer outcome. This approach is particularly pertinent at the 2-week and 6-week time points, when answering function-related questions such as walking, as post-operative clinical instructions would have been to remain non-weight-bearing. This is not an anticipated concern for the primary outcome, being at 12 weeks, nor the subsequent follow-ups.

Objective measures are included at the 2-week and 6-week time points: non-weight-bearing dorsiflexion and plantar flexion ROM and, ankle (figure-of-8) and calf (circumference) measures. Participants will be invited to attend for repeated outcome assessments at 12 weeks, 24 weeks and 52 weeks, with the additions of weight-bearing dorsiflexion, walking speed, balance and isokinetic plantar-flexion strength. Those declining will be encouraged to complete and return the appropriate PROMs questionnaires.

### Participant timeline

**Fig. 1:**
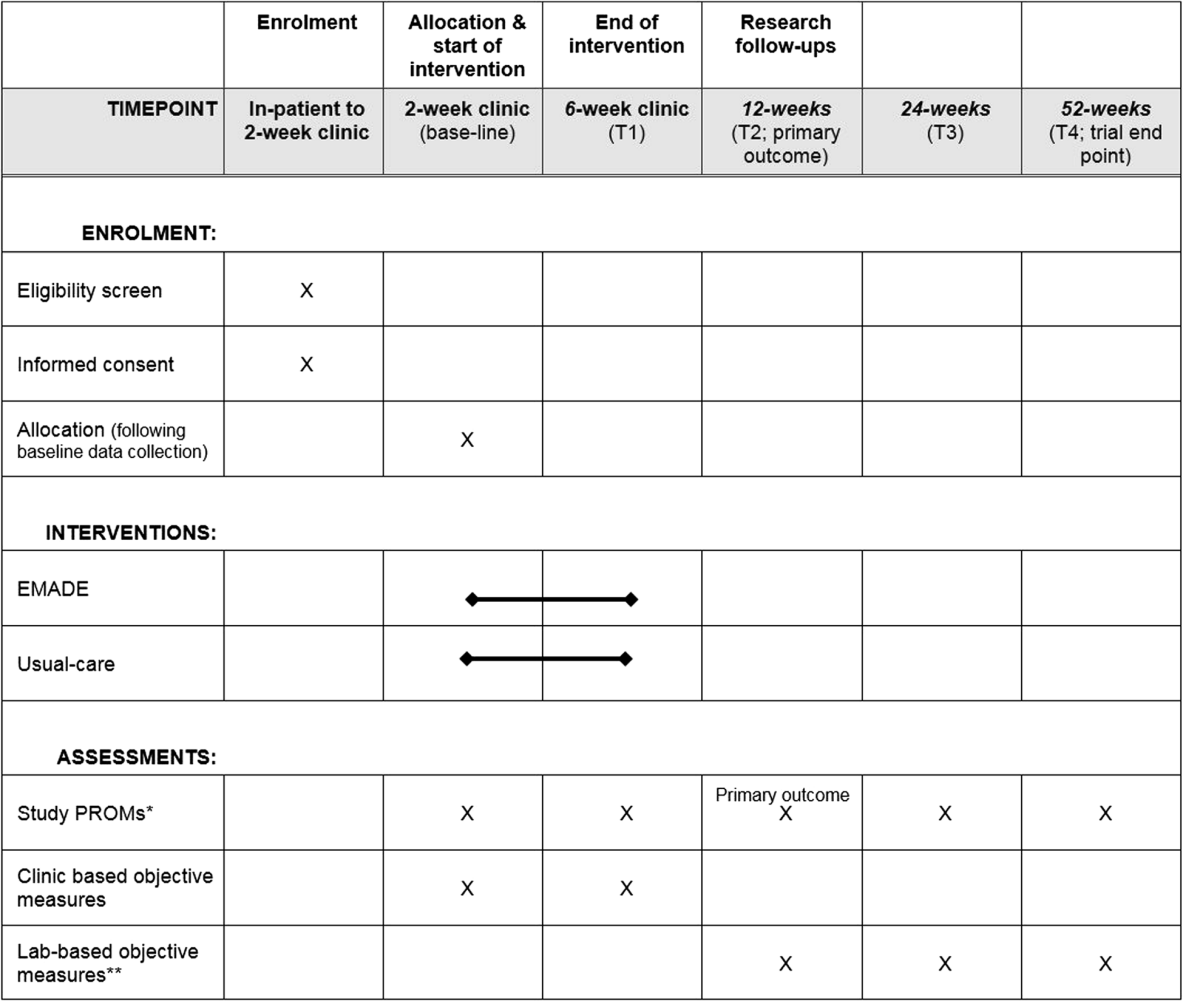
Standard Protocol Items: Recommendations for Interventional Trials (SPIRIT) Figure of enrolment, interventions and assessments. Key: *EMADE* early motion and directed exercise, *PROMs* Patient-reported outcome measures. *Study PROMs: Olerud Molander Ankle Score (OMAS), Ankle-Fracture Outcome of Rehabilitation Measure (A-FORM), EQ-5D-5 L, work and leisure activities, walking aid use and return to driving. **Option for patients to attend for Laboratory-based objective measures

**Fig. 2:**
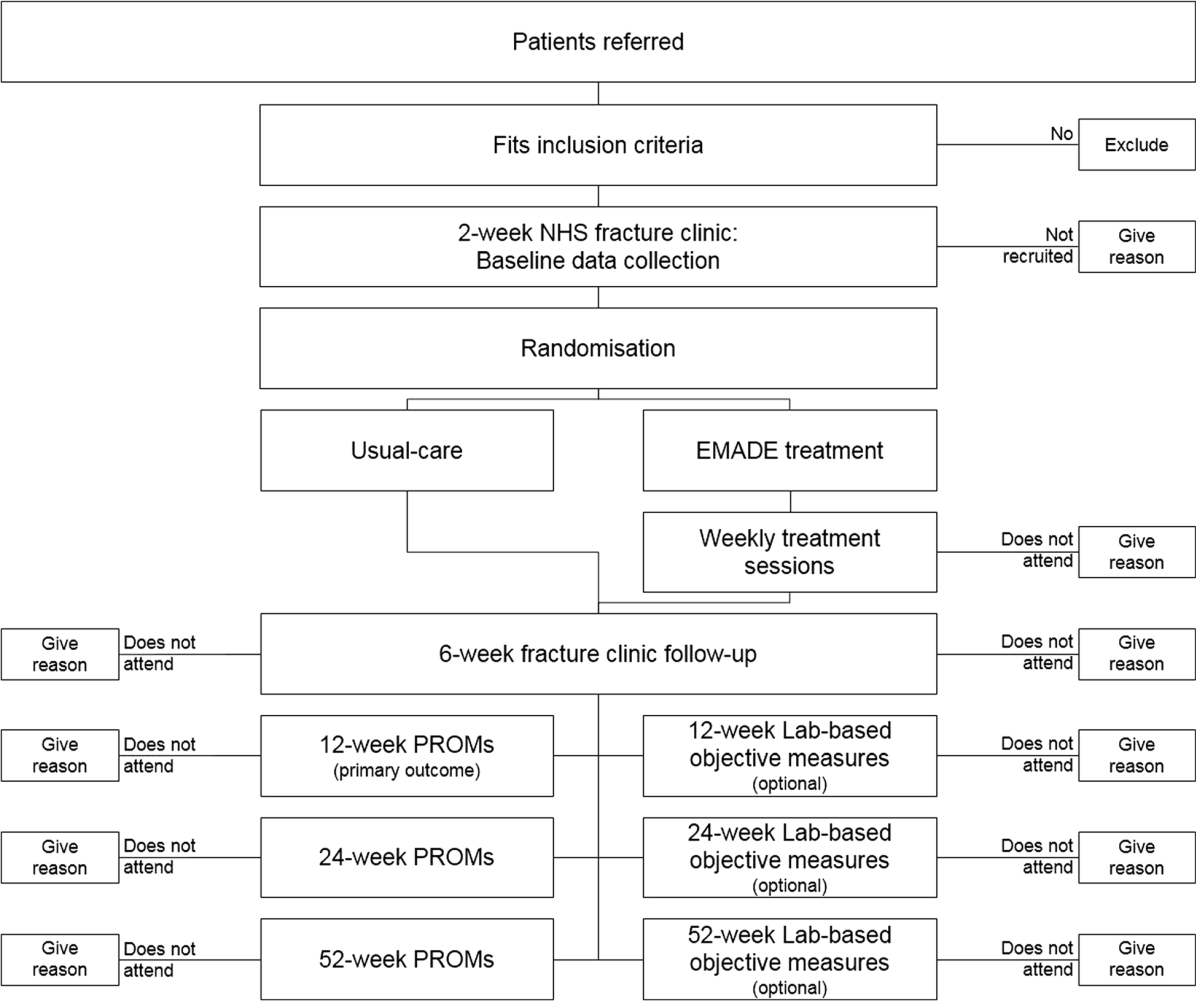
A schematic diagram of the patient’s journey through the early motion and directed exercises (EMADE) ankle study. Key: *EMADE* early motion and directed exercise, *PROMs* patient-reported outcome measures

Recruitment and consent may take place from the in-patient stay until and including the 2-week post-surgery outpatient review. Only after all baseline outcome measures are recorded, does randomisation take place, see Figs. 1 and 2.

The data collection time points (post surgery) are: baseline at 2 weeks, end of intervention at 6 weeks, and at three research follow-up time points; 12 weeks and 24 weeks as short- and mid-term follow-ups, respectively, and at 52 weeks as a long-term follow-up and the trial end point. For participant’s convenience, the 2-week and 6-week EMADE sessions are combined with the orthopaedic 2-week and 6-week reviews. The 12-week, 24-week and 52-week PROMs data may be collected via either return post (postage pre-paid), electronically or in person according to participant choice. Participants undergoing objective assessment will attend the David Greenfield Human Physiology Unit, Medical School, University of Nottingham, UK.

### Allocation and Blinding

An online computer service, www.sealedenvelope.com is used for randomisation. This facility generated the codes concealed from the research team, on a 1:1 allocation basis to either group, with random order of permuted blocks (sizes of 4, 6 and 8). Over the duration of the trial, there is greater potential that those with syndesmosis screw(s) may require further surgery. To mitigate against risk of allocation imbalance, stratification of this sub-group will be designed into the randomisation.

### Blinding (masking)

It is not possible to blind the participant nor the therapist from the treatment allocation.

However, to reduce risk of bias, 12-, 24- and 52-week paper PROMs are completed by participants, concealed from the researchers and stored in sealed opaque envelopes. A third party, blinded to the intervention group inputs the data. The electronic PROMs are web-based, held by Bristol Online Survey (BOS), www.onlinesurveys.ac.uk. Via this system at the appropriate time points, participants are e-mailed a single-use link to their e-PROM.

## Sample size, data processing and statistical methods

### Sample size calculations

It was estimated that a total of 120 participants will be required based on a minimal clinical important difference of 10 points on the OMAS, with a standard deviation of 19.5, significance level of 5% and powered at 80%. Allowance for attrition was set initially at 20% but, due to higher-than-expected dropout rates, this was increased to 30%, yielding a target of 156 to enter the study at randomisation.

### Data processing

PROM data collection is paper based, with participant number 55 onwards being offered the option of completing PROMs online. Once paper-based 12-week, 24-week and 52-week PROMs are completed the participant seals them in a windowless opaque envelop and returns them to the researchers (postage pre-paid). These are securely stored and subsequently opened in batches by an individual blinded to group allocation, who inputs the data into a holding database for subsequent transfer into SPSS for reporting and statistical analysis. For audit purpose, after inputting each batch, a ‘read-only’ copy is stored in a separate protected folder. A separate copy is used for data screening and cleaning, guided by good practice [33] and may include self-evident corrections [34] where appropriate. While filters and error alerts will reduce inputting errors, 20% of entries will be cross-checked against the paper PROMs for discrepancies. Data changes will be recorded for blinded adjudication. For audit purposes a copy of the cleaned and checked data will also be saved as a ‘read-only’ copy and stored separately. The design of the e-PROMs does not permit questions to be left unanswered, mitigating against problems of incomplete data and once the participant completes their PROM the original dataset is locked against further alterations. The system permits data transfer into SPSS.

The research team and statistician will conduct the analysis. The statistician will conduct the primary outcome analysis independently and is blinded to group allocation. Data and reason for any patient excluded prior to randomisation will be reported, but not carried into the main analysis. Descriptive statistics will be produced, within and between each intervention group(s) for demographics and outcomes at all data time points. Continuous data will be summarised as mean and standard deviation and confidence intervals (CI) (95% CI and *p* value threshold ≤ 0.05) for the PROM outcome data. Median and interquartile ranges will be used where appropriate statistical assumptions are not met, whereas categorical data will be presented as frequencies and proportions and analysed using either chi-square or Fisher’s exact test as indicated (*p* value of ≤ 0.05). Non-parametric tests will be employed where appropriate. Analysis will be conducted on the intention-to-treat (ITT) basis.

To test the primary outcome measure hypothesis that the EMADE physiotherapy intervention, as described will perform better than usual care, the difference in group means of the primary outcome, OMAS values at 12 weeks post surgery, will be statistically compared. This will be through applying an independent *t* test with significance taken as *p* value of ≤ 0.05 and the inclusion of confidence intervals (CI at 95% CI and *p* value of ≤ 0.05 threshold). Where data is not found to be normally distributed such that parametric tests cannot be utilised, the non-parametric Mann-Whitney *U* test will be employed (*p* value ≤ 0.05).

There is a lack of consensus in the academic literature on how to handle missing data [35, 36]. However, based on the 120 sample size, the following sequential approach will be applied for the primary outcome:

1. If missing data is 5% or less, conduct analysis based on complete-case analysis
2. If missing data is greater than 5% and up to 10%:
  - Conduct analysis based on pairwise deletion (assuming missing at random (MAR)) and then
  - Conduct multiple imputation analysis
  - If these two analyses yield comparable results, then it will be assumed that the missing data has not influenced the outcome of the trial
  - If the two analyses yield notable differences, to accommodate for the missing data, sensitivity analysis will be conducted based on the statistician’s advice
3. If missing data exceeds 10%, detailed discussions will be held with the study statistician

Interpretation of secondary outcome and sub-group analysis will require caution due to risks of type II errors and, because of where post hoc analysis may demonstrate association, causation may not be supported. Secondary analyses will be performed for hypothesis generation. The difference in group mean scores for the secondary outcomes; EQ-5D-5 L and A-FORM at 12 weeks post surgery will be analysed in a similar manner to the primary outcome. The one-way analysis of covariance (ANCOVA), including follow-up time as a covariance, will be applied to assess these outcomes across the complete study period.

Adjustment for baseline scores, through analysis of covariance, will be conducted for OMAS, EQ-5D-5 L and A-FORM and compared with unadjusted scores. Impact of covariates will be further explored through regression analysis. Initial analysis for co-variates where significance is nearly reached (*p* > 0.10) will be taken forward into a statistical model. Due to the bimodal impact of age and sex, these will be included in this analysis. Other exploratory analysis for potential covariates may include impact of smoking, previous level of physical activity, type of fracture (AO classification), complexity of surgical repair and any subsequent removal of metal work; most notably removal of syndesmosis screw(s) for which stratification was applied at randomisation. Analysis will also be conducted using modified ITT by excluding those participants who attend only one session or less of EMADE sessions.

Sub-groups will include return to work for those unable to work due to their ankle fracture at the time of randomisation, and similarly, return to driving for those unable to drive due to their ankle fracture at the time of randomisation. Frequency of complications will be described but, based on the published literature, it is anticipated that frequency will be sufficiently low not to warrant statistical analysis.

### Health economics

Health economic evaluations will be conducted from a societal perspective and from the service commissioner’s perspective. The societal perspective will be limited to incremental cost-effectiveness ratio (ICER) of the natural units of; return to work and return to driving (sub-groups as described earlier). Based on the EQ-5D-5 L PROM data, a cost-utility analysis (costs per quality-adjusted life year; QALY) will be performed from the local commission’s perspective. Sensitivity analysis will include the variability of the additional EMADE physiotherapy service costs; for example, £18 to £25 per session (2016 rates) and frequency of post-6-week physiotherapy sessions. The final methods for health economic evaluations will be guided by discussion with a health economist.

### Governance

Governance procedures were developed following Good Clinical Practice (GCP) guidelines (non-CTIMP) [37] with agreements from the local sponsors and the Ethics Committee. This includes reporting procedures for adverse and serious adverse events. The governance procedures for this trial has been independently audited by the Quality Assurance and Good Clinical Practice Audit Office, Nottingham Health Science Partnership, with a favourable outcome.

Due to the size of the trial it was considered unnecessary to form a data monitoring committee. This study will comply with the requirements of the UK Data Protection Act 1998 with regards to the collection, storage, processing and disclosure of personal information and will uphold the Act’s core principles. Access to collated participant data will be restricted to the research study staff. Allocation of a unique code to each participant will help to ensure confidentiality and anonymity. These identifiers will be used in all data, study material and reporting. Published results will not contain any personal data that could allow identification of individual participants. All electronic data will be stored on host NHS and University computers, with limited access under username and password protection system, as per host NHS and University ICT policies. Paper-based data will be stored in a locked filing cabinet in a key-coded room.

### Ethics approval and amendments

NRES Committee East Midlands – Nottingham 2, reviewed and approved this study on 4 November 2014 (14/EM/1213). The Ethics Committee and sponsors have been informed of important protocol changes and events. Amendments have focussed on an embedded study, which has subsequently been removed from the trial; only two participants were recruited to this embedded study and both participants withdrew from the trial during their in-patient stay and did not reach randomisation. Other amendments include: (1) expanding the recruitment period from the in-patient period, up to and including the 2-week clinical visit and (2) reducing the burden on the participant by decreasing the number of PROMs and permitting PROM completion online. The time point of randomisation, the intervention, and the primary and core outcome measures, have all remained unchanged since the start of recruitment.

### Dissemination

Routes of dissemination will include:

1. Medical and associated, conferences, meetings and journals
2. Patient participation seminars and meetings, such as those organised by Arthritis Research UK
3. Further data-sharing plans for the current study are unknown and will be made available at a later date

Decisions on authorship will be directed by guidelines from International Committee of Medical Journal Editors [38].

## Discussion

The aim of this trial is to determine if EMADE is more effective than usual care in reducing symptoms and restoring function in those who have undergone open reduction and internal fixation following a Weber B ankle fracture. While EMADE does not contain novel modalities, what is distinctive for this trial is EMADE’s early clinical application of a progressive ankle rehabilitation programme, being at 2 weeks post ankle surgery versus the traditional of starting after 6 weeks of immobilisation.

We do not anticipate significant bias from known confounders, such as age and sex, and unknown confounders, due to the limiting effect on bias from random allocation. However, as a pragmatic study, there is no control nor influence over the quality and volume of care, including physiotherapy, after the end of the intervention at 6 weeks post surgery.

While compliance with the EMADE home exercise programme will be encouraged through the weekly face-to-face reviews and the written and pictorial exercise sheets, compliance will still be a potential confounder. However, as a pragmatic trial, this level of encouragement and supervision was felt to be appropriate, as it reflects current physiotherapy clinical practice.

It may be argued that starting physiotherapy early with EMADE will reduce the number of sessions subsequently required and, thus, the overall number of sessions may not be significantly different from usual care. However, while this remains unproven it is pertinent to assume that EMADE is more expensive (approximately £54 to £75) and, therefore a cost-utility analysis in QALYs is required from the commissioner’s perspective to determine if EMADE is value for money. Also, while QALY units will be valued by commissioners, the use of natural units; return to driving and return to work will be tangible to patients and their families.

As this intervention requires no specialist equipment, it is suitable for provision in both primary and secondary care-based physiotherapy departments and, therefore, has potential for broad clinical impact. The trial design limits bias where possible, including during the collection, processing and analysis of PROM data. Objective outcomes will be recorded unblinded; however, being secondary outcomes and analysed separately from the PROM data, the potential impact of bias from objective data will have limited impact on the overall quality of the study.

The primary objective of this study is to test whether the EMADE intervention is effective in the clinical setting. Therefore, it is appropriate to assess effectiveness of EMADE against usual care in the clinical setting, through a p-RCT. If significant clinical benefits and cost-effectiveness are demonstrated, EMADE should become a viable treatment option. A larger-scale, multicentre trial may be required to influence national guidelines.

### Trial status

At the time of manuscript submission 111 participants had joined the trial.

### Protocol version

Version 2.2, 18 April 2017. First recruit June 2015, last expected mid 2018.

## Additional files

Additional file 1: Early motion and directed exercises (EMADE) Standard Protocol Items: Recommendations for Interventional Trials (SPIRIT) Checklist. (DOCX 53 kb)

Additional file 2: Early motion and directed exercises (EMADE) and Template for Intervention Description and Replication (TIDieR) Checklist. (DOCX 30 kb)

## Abbreviations

A-FORM: Ankle-Fracture Outcome of Rehabilitation
AO: Arbeitsgemeinschaft für Osteosynthesefragen
ARUK: Arthritis Research UK
CPAQ: Clinical Physical Activity Questionnaire
EMADE: Early motion and directed exercises
EQ-5D-5 L: EuroQol 5 Dimension 5-Level Questionnaire
ICER: Incremental cost-effectiveness ratio
OMAS: Olerud and Molander Ankle Score.
ORIF: Open reduction and internal fixation
p-RCT: Pragmatic randomised controlled trial
PROM: Patient-reported outcome measure
ROM: Range of motion
